# Neuroanatomical Basis for the Orexinergic Modulation of Anesthesia Arousal and Pain Control

**DOI:** 10.3389/fncel.2022.891631

**Published:** 2022-04-26

**Authors:** Xuaner Xiang, Yuzhang Chen, Ke-Xin Li, Jianqiao Fang, Philip E. Bickler, Zhonghui Guan, Wei Zhou

**Affiliations:** ^1^Department of Anesthesia and Perioperative Care, University of California, San Francisco, San Francisco, CA, United States; ^2^Department of Physiology, University of California, San Francisco, San Francisco, CA, United States; ^3^Key Laboratory of Acupuncture and Neurology of Zhejiang Province, The Third Clinical Medical College, Zhejiang Chinese Medical University, Hangzhou, China

**Keywords:** orexin, hypocretin, light sheet microscopy, optogenetics, anesthesia, analgesia

## Abstract

Hypothalamic orexin (hypocretin) neurons play crucial roles in arousal control. Their involvement in anesthesia and analgesia remains to be better understood. In order to enhance our view on the neuroanatomy, we systematically mapped the projections of orexin neurons with confocal microscope and light sheet microscope. We specifically expressed optogenetic opsins tagged with fluorescence markers in orexin neurons through adeno-associated viral infection in the mouse brain. The imaging results revealed fine details and novel features of the orexin projections throughout the brain, particularly related to the nuclei regulating arousal and pain. We then optogenetically activated orexin neurons in the lateral hypothalamus to study the effects on anesthesia-related behaviors. cFos staining showed that optogenetic stimulation can activate orexin neurons in the ChR2-mCherry group, but not the control mCherry group (62.86 ± 3.923% vs. 7.9 ± 2.072%; *P* < 0.0001). In behavior assays, optogenetic stimulation in the ChR2-mCherry group consistently elicited robust arousal from light isoflurane anesthesia (9.429 ± 3.804 s vs. 238.2 ± 17.42 s; *P* < 0.0001), shortened the emergence time after deep isoflurane anesthesia (109.5 ± 13.59 s vs. 213.8 ± 21.77 s; *P* = 0.0023), and increased the paw withdrawal latency in a hotplate test (11.45 ± 1.185 s vs. 8.767 ± 0.7775; *P* = 0.0317). The structural details of orexin fibers established the neuroanatomic basis for studying the role of orexin in anesthesia and analgesia.

## Introduction

Orexin, also known as hypocretin, was discovered in 1998 (orexin, from *orexis*, Greek for appetite; hypocretin, from the hypothalamus and homologous to gastrointestinal hormone secretin) (Gautvik et al., 1996; de Lecea et al., 1998; Sakurai et al., 1998). Orexin neurons were found to have a variety of roles in sleep-wake control, feeding, autonomic, neuroendocrine homeostasis, thermoregulation, and memory formation (Date et al., 1999; Zhang et al., 2005; Belanger-Willoughby et al., 2016; Xiao et al., 2021; Maski et al., 2022). Two homologous neuropeptides, orexin A (33 amino acids) and orexin B (28 amino acids) are derived from cleavage of the same prepro-orexin polypeptide (130 amino acids). They interact with two G-protein coupled receptors with different affinities and functions. While the cell bodies are localized in the tuberal part of the lateral hypothalamus, orexin fibers were found in many parts the brain and the spinal cord with a variety of functions (Peyron et al., 1998; van den Pol, 1999; Korotkova et al., 2003; Yang et al., 2019; Barr et al., 2021; Seoane-Collazo et al., 2021). To name a few, the projection to the basal forebrain promotes wakefulness and motivation (Wang D. et al., 2021), the projection to the lateral habenula is involved in the regulation of aggressive behavior in male mice (Flanigan et al., 2020), and the projection to the ventral tegmental area (VTA) has a role in regulating stress-induced cocaine recurrence (Tung et al., 2016). More work is needed to decipher the neuroanatomy of the orexin projections, which is a key step in understanding the various roles of orexin.

Although general anesthesia has advanced tremendously since the demonstration of ether in 1846, its mechanisms remain largely elusive (Eger et al., 2014). A number of neural circuits are involved in sleep/wake regulation, likely also linked to anesthesia mechanisms, such as histaminergic neurons in tuberomammillary nucleus (Kiyashchenko et al., 2002; Saper et al., 2010), noradrenergic neurons in locus coeruleus (Carter et al., 2012, 2013), serotonergic neurons in raphe nucleus (Liu et al., 2002; Yang et al., 2019), and cholinergic neurons in basal forebrain (Xu et al., 2015). Additionally, orexin neurons play crucial role in the modulation of sleep/wake cycle and central pain processing. A mutation in the canine orexin receptor leads to narcolepsy with the fragmentation of the sleep architecture, as well as cataplexy with sudden onset of muscle atonia (Lin et al., 1999). Some human narcoleptic patients have undetectable levels of orexin in their cerebrospinal fluid (Nishino et al., 2000). These patients may experience prolonged delay in anesthesia recovery and are more likely to have chronic pain (Hershner et al., 2019; Haack et al., 2020). Furthermore, animal experiments show that the orexin is involved in anesthesia arousal control, upper airway patency, autonomic tone, and gastroenteric motility, all of which are important aspects in anesthesia management (Kelz et al., 2008; Dong et al., 2009; Bulbul et al., 2010; Zhou et al., 2018; Olsen et al., 2021; Wang D. et al., 2021; Wang X. et al., 2021, p. 1). Orexin indeed plays crucial role in anesthesia. However, the understanding of the underlying mechanism is still insufficient.

In our previous study, we utilized the Designer Receptors Exclusively Activated by Designer Drugs (DREADD) to study the effects on anesthesia emergence and pain control (Zhou et al., 2018). To further study the orexin circuit in higher resolution, we applied optogenetics to investigate the details of the involvement of orexin neurons in anesthesia. Earlier works, using antibodies recognizing orexin peptides, laid the foundation on mapping the orexin neuronal projections (Peyron et al., 1998). In this study, we applied modern microscopy such as confocal and light sheet microscopies, and viral tracing technology to systematically map the 3D orexin projections in details. With the help from transgenic animals and viral technology (Zhang et al., 2010; Roth, 2016; Haggerty et al., 2020; Lanciego and Wouterlood, 2020), we found the expression of opsins in neurons through adeno-associated virus (AAV) not only provided an excellent tool to image the neuronal projections, but also enabled us to manipulate the activity of orexin neurons to study the functional effects on anesthesia-related behaviors.

## Materials and Methods

### Animals

We used male transgenic orexin-Cre C57BL/6 mice (8∼12 weeks old, weight 25–30 g) with Cre recombinase and EGFP under the prepro-orexin promoter, a gift from Dr. Akihiro Yamanaka, Nagoya University, Japan (Inutsuka et al., 2014). All experimental procedures involving animals were approved by the Institutional Animal Care and Use Committee, University of California, San Francisco. Mice were maintained in a strictly controlled environment with *ad libitum* access to food and water. The light cycle starts at 6:00 a.m. and ends at 6:00 p.m. and the dark cycle runs from 6:00 p.m. to 6:00 a.m. The temperature was controlled between 20 and 22^°^C. Altogether 72 mice were used in the experiments for imaging, electrophysiology, and optogenetic behavior assays.

### Virus Injections and Optical Fiber Implantation

Three different Cre-dependent viruses, AAV-Syn-DIO-ChrimsonR-tdTomato (62723-AAV5; Addgene), AAV-EF1α-DIO-hChR2(H134R)-mCherry-WPRE-HGHpA (20297-AAVretro; Addgene), and AAV-hSyn-DIO-mCherry (50459-AAV8; Addgene) were used in this study. Hereafter we refer to them as AAV5-DIO-ChrimsonR-tdT, AAVretro-DIO-ChR2-mCherry, and AAV8-DIO-mCherry. The titers of virus were 5 × 10^12^ vg/mL (AAV5-DIO-ChrimsonR-tdT), 1 × 10^13^ vg/mL (AAVretro-DIO-ChR2-mCherry and AAV8-DIO-mCherry). The viruses were aliquoted and stored at –80^°^C.

Intracranial injections were performed under balanced anesthesia using a robot stereotaxic instrument (Neurostar, Germany). Ketamine (80 mg/kg, i.p. injection), meloxifen (5 mg/kg, s.c. injection), ampicillin (10–20 mg/kg, s.c. injection), buprenorphine (0.1 mg/kg, s.c. injection), 0.25% bupivacaine (0.025 ml, s.c. injection) and isoflurane (Henry Schein Animal Health) were used. A 5 μl glass syringe with a 33 gauge needle (syringe: 87,943, needle: 7762–06; Hamilton Company) was used for the injections, and 0.5 μl virus was delivered over a 10 min (0.05 μl/min) period. The needle was left *in situ* for 10 min and slowly removed. The skin was stapled after surgery.

The injection coordinates were as follows: lateral hypothalamus (AP, –1.46 mm, ML, ± 0.95 mm, and DV, 5.1–5.0 mm), medial amygdala nucleus (AP, –1.94 mm, ML, ± 2.3 mm, and DV, 4.76–4.66 mm), medial preoptic area (AP, –0.1 mm, ML, ± 0.5 mm, and DV, 5.0–4.9 mm), lateral habenula (AP, –1.7 mm, ML, ± 0.44 mm, and DV, 3.2–3.1 mm), periaqueductal gray (AP, –4 mm, ML, ± 0.3 mm, and DV, 3.2–3.1 mm), and locus coeruleus (AP, –5.34 mm, ML, ± 0.85 mm, and DV, 4.5–4.4 mm).

For optogenetic stimulation experiments, all mice were implanted with bilateral optical fibers (400 μm Core, 0.39 N.A.; R-FOC-BL400C39NA; RWD Life Science) into the lateral hypothalamus (AP, –1.46 mm, ML, ± 0.95 mm, and DV, 5.0 mm).

### Electroencephalogram/Electromyography Implantation

One week after receiving bilateral stereotaxic viral injections, the animals were subjected to optical fiber implantation and electroencephalogram (EEG)/electromyography (EMG) device implantation (Pinnacle Technologies). A 23-gauge surgical needle was used to drill four guide holes, including two frontal cortical area (AP, 1 mm, ML, ± 1.25 mm) and two parietal area (AP, –3 mm, ML, ± 2.5 mm). Four screws with wires were placed into the skull through the holes. The wires were then soldered onto a six-pin connector EEG/EMG headstage. EMG leads were placed into the neck muscle. The headstage was secured with dental cement (C&B Metabond^®^ Quick Adhesive Cement System, Parkell) onto the skull. Behavioral experiments were conducted 2 weeks later after surgery to allow for sufficient recovery. Each time before the behavioral tests, mice were anesthetized with 3% isoflurane for 1 min in an induction chamber followed by connecting with the EEG device.

### Immunohistochemistry

The mice were transcardially perfused 3–4 weeks after virus injection with 1X cold PBS followed by a cold solution of 4% paraformaldehyde (PFA, Electron Microscopy Services) in PBS. Brains were removed and post-fixed in 4% PFA for 6 h at 4°C before being transferred to 30% sucrose for at least 2 days of dehydration until the tissue stayed afloat. A series of 50 μm coronal/sagittal brain slices were sliced using a cryostat (Leica CM3050S). Fixed brain slices were used for immunohistochemistry staining.

Slices were blocked with blocking solution (5% donkey serum, 3% BSA, and 0.3% Triton-X100 in PBS) for 1 h at room temperature, followed by overnight incubation with primary antibodies at 4^°^C for 18 h. After the brain slices were washed three times with PBS, 10 min each time, the slices were incubated with secondary antibodies for 2 h at room temperature followed by 3 washes. The slices were covered with DAPI Fluoromount-G mounting medium (0100-20, SouthernBiotech).

*The antibodies were diluted in blocking solution as follows.*

mCherry. Primary, chicken anti-mCherry (1:400, NBP2-25158, Novus Biologicals); rabbit anti-mCherry (1:100, ab167453, Abcam). Secondary, Cy3-conjugated AffiniPure goat anti-chicken IgY++ (1:400, 103-165-155, Jackson Labs); goat anti-rabbit Cy3 (1:100, 111-165-003, Jackson Labs).

cFos. Primary, rabbit anti-cFos (1:100, 2250S, cell signaling). Secondary, Alexa Fluor 488-conjugated donkey anti-rabbit IgG (H + L) (1:400, 711-546-152, Jackson Labs).

Orexin. Primary, mouse anti-Orexin-A (1:100, sc-80263, Santa Cruz). Secondary, Alexa Fluor 488-conjugated donkey anti-mouse IgG (H + L) (1:400, 715-545-151, Jackson Labs).

### Imaging

Confocal images were taken with the Leica TCS SP8. Light sheet images were taken with ZEISS Lightsheet Z.1. Lightsheet images were collected using EC Plan-Neofluar 5x NA 0.16 objective. The pixels are 1,920 × 1,920, and the image size is 4867.7 μm × 4867.7 μm. Tile scans are assisted with the setup of a tiling experiment with Zen (ZEISS) for Lightsheet Z.1 software. Tiles were stitched and 3D video was generated by Imaris software (OXFORD INSTRUMENTS). ImageJ was used to visualize the 3D images.

### Brain Tissue Clearing

Animal was transcardially perfused using 15 ml ice-cold 1X PBS (3 ml/min for 5 min), followed by 20 ml of PFA-Monomer hydrogel solution (1.3% acrylamide, 0.0125% Bis-acrylamide, 4% PFA, 0.25% VA-044 initiator (w/v) in 1X PBS solution (capture-clarity.org/optimized-clarity). The brain was cut into two hemispheres and placed into hydrogel solution for 3 days for monomer diffusion at 4^°^C in the cold room. Afterward, the hemispheres were transferred to a 50 ml conical tube, which was placed in a vacuum chamber. The tube was shaken gently at 37^°^C for 24 h for polymerization. The hemispheres were transferred into 200 mM NaOH-Boric buffer (pH 8.5), 8% SDS for 6–12 h with gentle shaking to remove residual PFA. Next, they were cleared in 100 mM Tris-Boric buffer (pH 8.5), 8% SDS with gentle stirring at 37^°^C over the course of 21 days to remove the phospholipids. The cleared hemispheres were then washed in 1X PBST (0.1% Triton-X100) for 24 h to remove SDS. Thereafter, one hemisphere was stained with the primary antibody for 6 days at 37°C (2 mL of PBST + rabbit anti-mCherry, 1:100 dilution). After washing in 1X PBST, the hemisphere was stained in 1:100 dilution of goat anti-rabbit Cy3 at 37^°^C for 5 days. And then washed the hemispheres extensively with 1X PBST at 37^°^C for 3 days with solution change twice a day. Before imaging, the hemispheres were placed in the EasyIndex solution (Lifecanvas, refractive index—1.46) for 3 days at room temperature.

### Patch-Clamp Recording From Brain Slices

AAVretro-DIO-ChR2-mCherry was injected into the lateral hypothalamus of orexin-Cre mice 3–4 weeks prior to electrophysiological experiments. For brain slice preparation, mice were anesthetized with isoflurane and transcardially perfused with NMDG-HEPES: 93 mM NMDG, 93 mM HCl, 2.5 mM KCl, 1.2 mM NaH_2_PO_4_, 30 mM NaHCO_3_, 20 mM HEPES, 25 mM Glucose, 5 mM sodium ascorbate, 2 mM Thiourea, 3 mM sodium pyruvate, 10 mM MgSO4.7 H_2_O, 0.5 mM CaCl2.2H_2_O (pH 7.4 by HCl, 300–310 mOsm), bubbled with carbogen (95% O2/5% CO2). The coronal sections were cut into 300 μm thickness sections by a microtome (Leica VT1000). The brain slices were recovered in the cutting solution for 15 min at 34^°^C and in the artificial cerebrospinal fluid (aCSF) recovery solution for 45 min at room temperature before recording. The aCSF solution contained: 125 mM NaCl, 26 mM NaHCO3, 1.25 mM NaH_2_PO_4_, 2.5 mM KCl, 1 mM MgCl2, 2 mM CaCl_2_, 12.4 mM glucose, 300–310 mOsm, and bubbled with carbogen. The final pH was 7.4.

Loose patch recordings were made on mCherry-positive cell bodies identified under the fluorescence microscope. The internal solution within whole-cell recording pipettes (3–5 MΩ) contained: 139.95 mM KGluc, 8.5 mM KCl, 10 mM HEPES, 0.1 mM EGTA, 4 mM MgATP2, 0.3 mM NaGTP (pH 7.3–7.4, 300–310 mOsm). During the recordings, the neurons were perfused with the carbogen-bubbled aCSF solution with a peristaltic pump. The recordings were performed using a Multiclamp 700B amplifier (Molecular Devices). The data were acquired and analyzed using pClamp and ClampEx software (Molecular Devices). For light stimulation, 470 nm LED (M470F3, Thorlabs) was used to deliver light pulses (1, 5, 10, 20 Hz, 10 ms pulse width). The optical fiber was directed right above the patched cell with a micromanipulator (Sutter Instrument) under the recording microscope.

### Isoflurane Arousal Test

The animals were anesthetized with 3% isoflurane for 1 min in an induction chamber and then quickly attached with optical fibers. Then the mouse was placed in a clear isoflurane chamber with optical fibers exiting through a sealed port. The chamber was then equilibrated with 0.75% isoflurane for 10 min, before light pulse (473 nm, 20 Hz, 20 ms pulse width, 30 s, Aurora-200, Newdoon) was applied to the animals. After the 30 s stimulation, isoflurane was continued for another 1 min before it was switched to 100% O_2_ to let the mouse recover. The time from starting the light pulse to the point when the mouse started to wake up (moving, kicking, or tail rising) was recorded as the latency to wake. If animals had no response to the light stimulations, the time from laser on to when the animals woke up after the termination of isoflurane was recorded as latency to wake. All anesthesia chambers were placed on a temperature-controlled heating pad to automatically regulate the temperature between 35 and 37^°^C. The trials were repeated three times in the same day from 9:00 to 15:00, and again repeated two more times with 3-day intervals. Mice with AAV8-DIO-mCherry injections were used as control and received the same treatment of optical stimulation. All trials were age-matched with similar animals.

### Isoflurane Emergence Test

The experimental animals received 2% isoflurane for 30 min in the induction chamber. Afterward, the animals were quickly attached with optical fibers and then were treated with or without optical stimulation (20 Hz, 20 ms pulse width, 1 s on, 1 s off) in a clear acrylic chamber open to the room air while placed on their backs to test how quickly the righting reflex can return (RoRR). The RoRR, when the animal turned around and stood on all four paws, was used as the indicator for the emergence from anesthesia. The emergence time from turning off the isoflurane to the RoRR was recorded for analysis. The trials were performed from 9:00 to 15:00 and repeated three times at 3-day intervals. The isoflurane induction chamber was placed on a temperature-controlled heating pad and a temperature probe connected to a temperature controller was placed underneath the animal body to automatically regulate the temperature between 35 and 37^°^C. Mice with AAV8-DIO-mCherry injections were used as control and received the same treatments. All trials were age-matched with similar animals.

### Hot Plate Test

The animals were anesthetized in the induction chamber with 2% isoflurane for 1 min to facilitate optical fiber attachment. Then the animals went through 5 min of optical stimulation with or without the laser (20 Hz, 20 ms pulse width, 1 s on, 1 s off), followed by the hot plate test. An acrylic container was used as an enclosure to prevent the animal from escaping. The temperature used in the hot plate test was 55^°^C. The animals were placed onto the hot plate while continuing the optical stimulation until the first time when animals showed licking, fanning, or jumping, at which point the animal was immediately removed from the hot plate and the paw withdrawal latency was recorded. The animal was removed if there was no response within 45 s. The trials were repeated three times in the same day from 9:00 to 15:00, with 60 min intervals, and again repeated two more times with 3-day intervals. Mice with AAV8-DIO-mCherry injections were used as control and received the same treatments. All trials were age matched with similar animals.

### Statistics

Data analysis was done by experimenters blinded to experimental conditions. Paired *t*-test was used to analyze the significance between the same group of mice with and without optogenetic stimulation. Unpaired *t*-test was used to analyze the significance of the data from two groups. Data are shown as mean ± s.e. of the mean (s.e.m.). Statistical significance was set at *P* < 0.05. All data were analyzed using Prism 7.0 (GraphPad Software Inc. San Diego, CA, United States).

## Results

### Specific Expression of Opsins in Orexin Neurons

**FIGURE 1:**
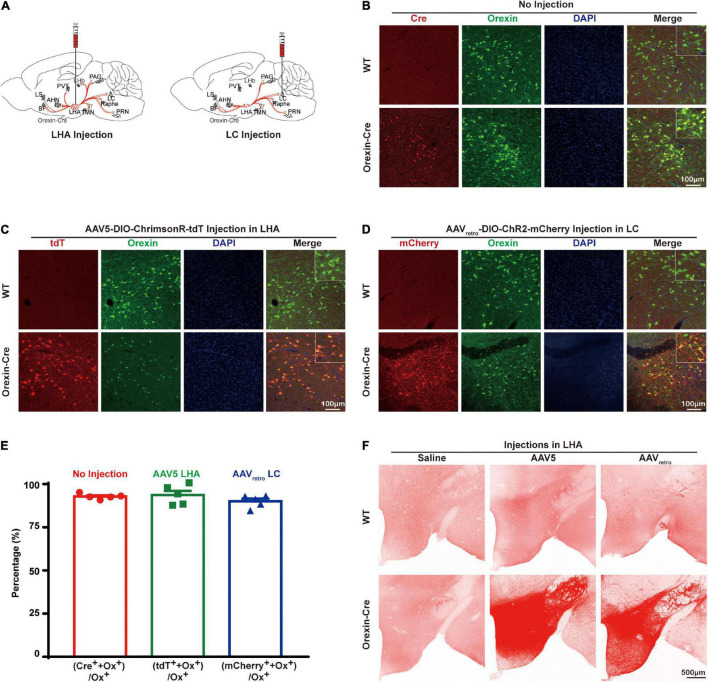
Opsin expression in the orexin-Cre mouse brain. **(A)** Schematic diagrams illustrate AAV virus injections into the orexin-Cre mouse brain. **(B)** Immunostaining of Cre (red), orexin (green), and DAPI (blue) in brain slices showed an overlapping expression of Cre and orexin in only orexin-Cre mice but not wildtype (WT) mice. **(C,D)** To express opsin in orexin neurons, orexin-Cre or WT mice received injections of AAV5-DIO-ChrimsonR-tdT into the LHA **(C)** and AAVretro-DIO-ChR2-mCherry into the LC **(D)**, and the brain slices were stained for mCherry (red), orexin (green), and DAPI (blue). The result showed expression of ChrimsonR-tdT and ChR2-mCherry in orexin neurons in orexin-Cre mouse but not WT mouse. **(E)** Quantitative analysis showed 92.87 ± 0.6974% (*n* = 5) colocalization of Cre recombinase with orexin in no-injection group, 93.7 ± 2.456% (*n* = 5) colocalization of tdTomato with orexin in AAV5 group, and 90.07 ± 1.593 % (*n* = 5) colocalization of mCherry with orexin in AAVretro group. **(F)** To compare the expression levels from different AAV serotypes, expressions in hypothalamus from saline, AAV5-DIO-ChrimsonR-tdT, and AAVretro-DIO-ChR2-mCherry injections in the WT or orexin-Cre mice are shown. AHN, anterior hypothalamus nucleus; BF, basal forebrain; LC, locus coeruleus; LHA, lateral hypothalamus area; LHb, lateral habenula; LS, lateral septum; PAG, periaqueductal gray; PVT, paraventricular nucleus of the thalamus; PRN, pontine reticular nucleus, TMN, tuberomammillary nucleus.

To express opsins in orexin neurons specifically, AAV5-DIO-ChrimsonR-tdT or AAVretro-DIO-ChR2-mCherry was injected into orexin-Cre mouse brains (Figure 1A). We stained the brain slices with antibodies recognizing orexin and Cre, which showed that Cre is colocalized with orexin in the lateral hypothalamus in orexin-Cre mice but no Cre was found in wildtype (WT) mice, suggesting that Cre was exclusively expressed in orexin neurons with colocalization of 92.87 ± 0.6974% (*n* = 5) (Figure 1B). Next, we injected AAV5-DIO-ChrimsonR-tdT into the lateral hypothalamus and AAVretro-DIO-ChR2-mCherry into the locus coeruleus. The brain slice imaging result showed 93.7 ± 2.456% (*n* = 5) colocalization of tdTomato with orexin in AAV5 group (Figures 1C,E), and 90.07 ± 1.593% (*n* = 5) colocalization of mCherry with orexin in AAVretro group in the lateral hypothalamus (Figures 1D,E). Interestingly, not all orexin neurons were mCherry positive, but all mCherry positive neurons were also orexin positive (Figure 1D), suggesting that not all of the orexin neurons in the lateral hypothalamus project to locus coeruleus. It’s noticeable that the processes are clearly visible with mCherry staining, but barely with orexin staining (Figure 1D), suggesting AAV expression of opsin tagged with fluorescence marker provided an excellent tool to label the neuronal projections. Lateral hypothalamus injections of both AAVretro-DIO-ChR2-mCherry and AAV5-DIO-ChrimsonR-tdT can produce similarly vigorous expression throughout the mouse brain (Figure 1F). In contrast, there was little signal in WT mice that received either AAV5 or AAVretro injections, similar to orexin-Cre mice with saline injections (Figure 1F).

### Projections of Orexin Neurons

**FIGURE 2:**
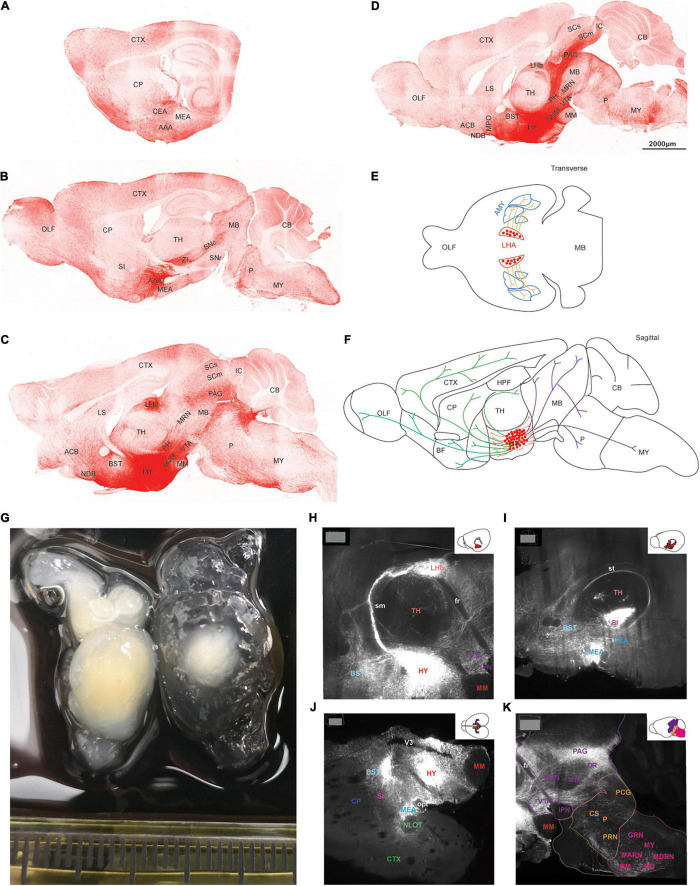
The topography of orexin projections. Orexin-Cre mice were injected with AAV5-DIO-ChrimsonR-tdT into LHA. **(A–D)** Representative confocal images from lateral to medial showing the orexin projections in sagittal planes. **(E,F)** Schematic drawings of lateral **(E)**, hypothalamic, rostral, and caudal **(F)** projections of orexin neurons. **(G)** Two hemispheres of mouse brain underwent tissue clearing for 1 week (left) and 3 weeks (right). **(H–K)** Orexin projections revealed by the lightsheet microscope. The images were chosen at particular angles to show certain interesting projection features. AAA, anterior amygdalar area; ACB, nucleus accumbens; AMY, amygdala; BST, bed nuclei of the stria terminalis; CB, cerebellum; CEA, central amygdalar nucleus; CP, caudoputamen; CS, superior central nucleus raphe; CTX, cerebral cortex; DR, dorsal nucleus raphe; fr, fasciculus retroflexus; GRN, gigantocellular reticular nucleus; HPF, hippocampal formation; HY, hypothalamus; IC, inferior colliculus; IPN, interpeduncular nucleus; LHb, lateral habenula; LS, lateral septal nucleus; MARN, magnocellular reticular nucleus; MB, midbrain; MEA, medial amygdalar nucleus; MM, medial mammillary nucleus; MPO, medial preoptic area; MRN, midbrain reticular nucleus; MY, medulla; NDB, diagonal band; NLOT, nucleus of the lateral olfactory tract; OLF, olfactory areas; opt, optic tract; P, pons; PAG, periaqueductal gray; PCG, pontine central gray; PH, posterior hypothalamic nucleus; PRN, pontine reticular nucleus; RM, nucleus raphe magnus; RO, nucleus raphe obscurus; SC, superior colliculus; SI, substantia innominata; sm, stria medullaris; SN, substantia nigra; st, stria terminalis; SUM, supramammillary nucleus; TH, thalamus; V3, third ventricle; VTA, ventral tegmental area; ZI, zona incerta.

**FIGURE 3:**
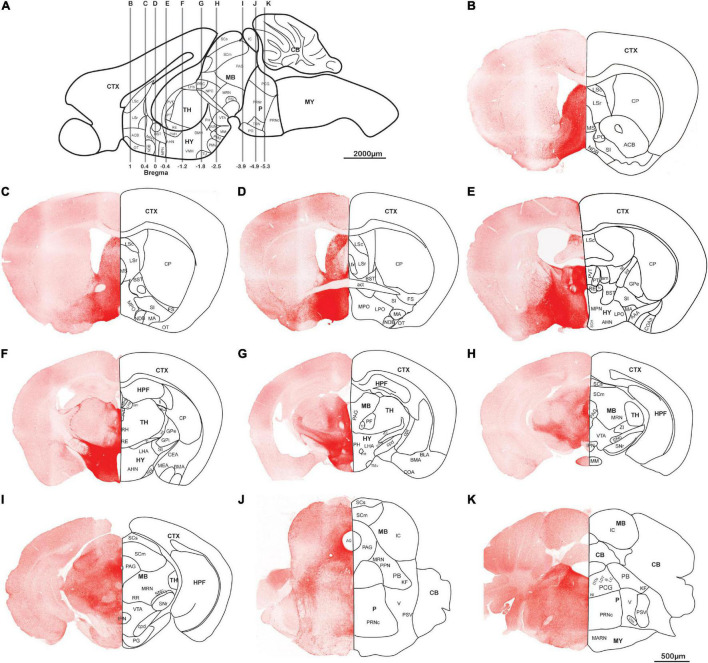
Orexin projections in coronal planes. Orexin-Cre mice were injected with AAV5-DIO-ChrimsonR-tdT into LHA. **(A)** Schematic diagram showing the anterior-posterior positions of the coronal brain slices. **(B–K)** Representative confocal images showing the orexin projections in coronal planes. Scale bars: 500 μm. AAA, anterior amygdalar area; act, anterior commissure, temporal limb; AHN, anterior hypothalamic nucleus; ACB, nucleus accumbens; AQ, cerebral aqueduct; B, barrington’s nucleus; BLA, basolateral amygdalar nucleus; BMA, basomedial amygdalar nucleus; BST, bed nuclei of the stria terminalis; CB, cerebellum; CEA, central amygdalar nucleus; COA, cortical amygdalar area; COAa, cortical amygdalar area, anterior part; CP, caudoputamen; cpd, cerebal peduncle; CTX, cerebral cortex; DMH, dorsomedial nucleus of the hypothalamus; DTN, dorsal tegmental nucleus; fi, fimbria; fr, fasciculus retroflexus; fx, columns of the fornix; FS, fundus of striatum; GPe, globus pallidus, external segment; GPi, globus pallidus, internal segment; HPF, hippocampal formation; HY, hypothalamus; int, internal capsule; IC, inferior colliculus; IPN, interpeduncular nucleus; KF, Koelliker-Fuse subnucleus; LC, locus coeruleus; LDT, laterodorsal tegmental nucleus; LHA, lateral hypothalamic area; LHb, lateral habenula; LPO, lateral preoptic area; LSc, lateral septal nucleus, caudodorsal part; LSr, lateral septal nucleus, rostroventral part; mtt, mammillothalamic tract; MA, magnocellular nucleus; MARN, magnocellular reticular nucleus; MB, midbrain; MEA, medial amygdalar nucleus; MHb, medial habenula; MM, medial mammillary nucleus; MPN, medial preoptic nucleus; MPO, medial preoptic area; MRN, midbrain reticular nucleus; MS, medial septal nucleus; MY, medulla; NI, nucleus incertus; NDB, diagonal band; NPC, nucleus of the posterior commissure; opt, optic tract; OT, olfactory tubercle; P, pons; PAG, periaqueductal gray; PB, parabrachial nucleus; PCG, pontine central gray; PF, parafascicular nucleus; PG, pontine gray; PH, posterior hypothalamic nucleus; PMd, dorsal premammillary nucleus; PMv, ventral premammillary nucleus; PPN, pedunculopontine nucleus; PRC, precommissural nucleus; PRNr, pontine reticular nucleus; PRNc, pontine reticular nucleus, caudal part; PSV, principal sensory nucleus of the trigeminal; PT, parataenial nucleus; PVT, paraventricular nucleus of the thalamus; PVH, paraventricular hypothalamic nucleus; RE, nucleus of reunions; RH, rhomboid nucleus; RN, red nucleus; RR, midbrain reticular nucleus, retrorubral area; sm, stria medullaris; st, stria terminalis; SCH, suprachiasmatic nucleus; SCm, superior colliculus, motor related; SCs, superior colliculus, sensory related; SI, substantia innominata; STN, subthalamic nucleus; SNc, substantia nigra, compact part; SNr, substantia nigra, reticular part; SUM, supramammillary nucleus; TH, thalamus; TMv, tuberomammillary nucleus, ventral part; TRN, tegmental reticular nucleus; TU, tuberal nucleus; V, motor nucleus of trigeminal; VIIn, facial nerve; VMH, ventromedial hypothalamic nucleus; VTA, ventral tegmental area; ZI, zona incerta.

AAV5-DIO-ChrimsonR-tdT was injected into the lateral hypothalamus of orexin-Cre mice, and sagittal (Figures 2A–D) and coronal (Figure 3) slices were imaged. Overall, strong tdTomato signals were observed in many subcortical regions, including the septum, basal forebrain, amygdala, hypothalamus, the superficial regions of thalamus, midbrain, pons, and medulla (Figures 2, 3, and Supplementary Table 1). Only sparse signals can be found in the olfactory area, caudoputamen, and cerebral cortex (Figures 2A–D, 3B–I). In contrast, several structures had little or no signals, including hippocampal formation, the core of thalamus, and cerebellum (Figures 2A–D, 3F–I). Interestingly, we found dense orexin fibers projecting to many sleep-wake and pain control nuclei (Supplementary Figure 1).

To visualize the three-dimensional (3D) orexin projections, we applied light sheet technology to image ChR2-mCherry in the clarified mouse brain (Figures 2G–K). Certain anatomical features, which were hard to recognize in the two-dimensional (2D) images by confocal, were more easily identifiable in the 3D light sheet rendering (Supplementary Figure 2 and Supplementary Video 1).

#### Hypothalamic Projections

Orexin neurons project intensely and diffusely in the hypothalamus except the mammillary body, which clearly had negative staining. The suprachiasmatic nucleus, the key pacemaker of the circadian rhythms in the body, was also noted to have significantly less fluorescence signals than the surrounding area (Figures 2C,D, 3D–G).

#### Rostral Projections

Rostrally, orexin fiber staining was found abundantly in bed nuclei of the stria terminalis (BST), basal forebrain (including the substantia innominata, diagonal band nucleus, and magnocellular nucleus), septum, and the superficial region of thalamus (Figures 2B–D, 3D–I). Many midline nuclei of thalamus were positive for orexin fibers, including paraventricular nucleus of thalamus (PVT), parataenial nucleus, nucleus of reuniens, rhomboid nucleus, central medial nucleus, anterodorsal nucleus, and intermedial dorsal nucleus. In particular, the lateral habenula, located in the dorsomedial part of the thalamus, has very dense staining of orexin fibers, in sharp contrast to the negatively stained medial habenula (Figures 2C,D,H, 3F–H). With the help of lightsheet data, the distinct fiber bundle from the lateral hypothalamus to the lateral habenula turned out to be stria medullaris (*sm*), clearly seen on the anteromedial surface of the thalamus. Posterior to the lateral habenula was the negative stained fasciculus retroflexus (*fr*) connecting the lateral habenula to the interpeduncular nucleus (Figure 2E, Supplementary Figure 2, and Supplementary Video 1).

#### Lateral Projections

Laterally, orexin fibers project extensively into the amygdala, particularly anterior, central, and medial amygdala area (Figures 2A,I, 3D–F). Near *sm*, stria terminalis (*st*), branched out from the BST and projected posterolaterally to the amygdala, especially to the central and medial amygdala nucleus (Figure 2I and Supplementary Video 1). Due to an oblique angle, the projection of *st* was not easily identified on orthogonal confocal images until it was revealed by the 3D light sheet image.

#### Caudal Projections

Caudally, along the posterior border of the thalamus, a distinct dense bundle of orexin fibers from posterior hypothalamus projected into the midbrain along the 3rd ventricle and aqueduct (Figures 2J, 3F–J). Other than interpeduncular nucleus and superior cerebellar peduncle, orexin fibers spread throughout the midbrain, particularly in the periaqueductal gray (PAG) region. A mesh network of orexin fibers was observed in multiple reticular nuclei in the midbrain, pons, and medulla. Several raphe nuclei were positive with orexin fibers throughout the brain stem, such as dorsal nucleus raphe, superior central nucleus raphe, nucleus raphe magnus, and nucleus raphe obscurus (Figures 2C,D,K, 3G–J).

The zona incerta, located in the posterior and lateral part of the hypothalamus, is densely innervated by orexin fibers. Nearby were some positively stained structures, including substantia nigra, VTA, midbrain reticular nucleus, globus pallidus, substantia innominata, and subthalamic nucleus (Figures 2B–D,K, 3F–H).

In the pons, orexin fibers had dispersed staining in most of the regions, with some highlights in the parabrachial nucleus, locus coeruleus, Barrington’s nucleus, laterodorsal tegmental nucleus, nucleus incertus and the superficial layer lining the 4th ventricle. The pontine reticular nucleus is covered by a meshed distribution of orexin fibers (Figures 2B–D,K, 3J–K).

In the medulla, orexin fibers were mostly distributed in the reticular nuclei, such as parvicellular, intermediate, gigantocellular, and magnocellular reticular nuclei (Figures 2B–D,K, 3K).

### Orexin Neuron Projects Both Rostrally and Caudally

To confirm the projection patterns above, AAVretro-DIO-ChR2-mCherry was injected into selected brain targets of orexin-Cre mice to see whether the retrograde expression can trace back to the cell bodies.

**FIGURE 4:**
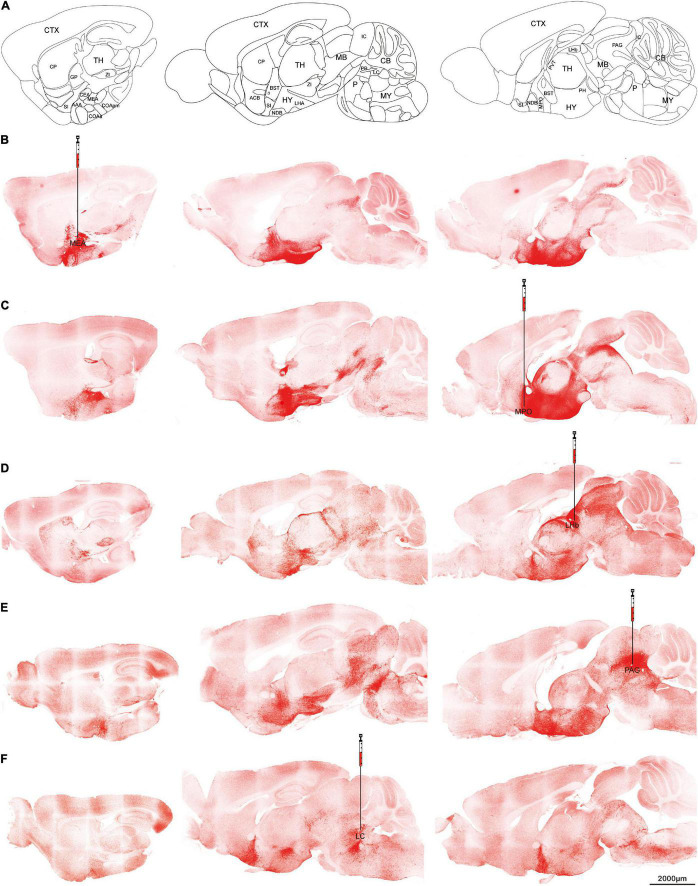
Retrograde tracing Orexin fibers. Orexin-Cre mice received retrograde AAVretro-DIO-ChR2-mCherry injections at different target sites. **(A)** Sagittal atlas diagrams corresponding to the confocal images. **(B–F)** Five different injection sites including MEA **(B)**, MPO **(C)**, LHb **(D)**, PAG **(E)**, or LC **(F)**. Scale bars: 2,000 μm. AAA, anterior amygdalar area; ACB, nucleus accumbens; BST, bed nuclei of the stria terminalis; CB, cerebellum; CEA, central amygdalar nucleus; COAa, cortical amygdalar area, anterior part; COApm, cortical amygdalar area, posterior part, medial zone; CP, caudoputamen; CTX, cerebral cortex; GP, globus pallidus; HY, hypothalamus; IC, inferior colliculus; LC, locus coeruleus; LHA, lateral hypothalamic area; LHb, lateral habenula; MB, midbrain; MEA, medial amygdalar nucleus; MPO, medial preoptic area; MY, medulla; NDB, diagonal band nucleus; P, pons; PAG, periaqueductal gray; PB, parabrachial nucleus; PH, posterior hypothalamic nucleus; PVT, paraventricular nucleus of the thalamus; TH, thalamus; SI, substantia innominata; ZI, zona incerta.

Five target sites were chosen for the retrograde virus injection. All of them produced robust expressions of ChR2-mCherry. Medial amygdala nucleus (Figures 4A,B), and PAG (Figure 4E) injections produced signals in the thalamic regions but much less than the injections from medial preoptic area (Figure 4C) or lateral habenula (Figure 4D). On the slices from lateral habenula injections, the mCherry signals were found not only in the thalamus, but also in anterior hypothalamus and midbrain (Figure 4D). Locus coeruleus injections showed the soma staining was concentrated at dorsolateral hypothalamus, and no expression was noted in the thalamus region including the lateral habenula (Figure 4F). Furthermore, we observed the rostral projection beyond the cell bodies to the anterior hypothalamus and BST regions. These differences implied that different orexin neurons have specific projection preferences but all orexin neurons seem to project both rostrally and caudally.

### Optogenetic Activation of Orexin Facilitated Arousal and Pain Tolerance

**FIGURE 5:**
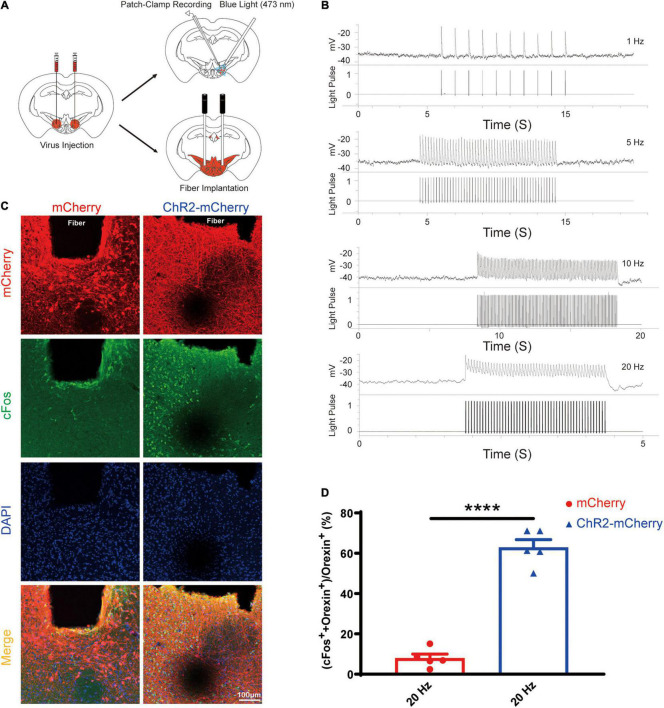
Optogenetic stimulation can reliably excite the orexin neurons expressing ChR2-mCherry. **(A)** Schematic diagram showing virus injection, patch-clamp recording, and optical fiber implantation. **(B)** Acute brain slices from mice expressing ChR2-mCherry were used for loose patch recordings. The results showed that orexin neurons expressing ChR2-mCherry can be reliably excited by blue light (473 nm) at different frequencies from 1 to 20 Hz. **(C)** Slices from mice expressing mCherry or ChR2-mCherry are stained for mCherry (red), cFos (green), and DAPI (blue) in lateral hypothalamus. **(D)** The statistical comparison of cFos staining in the mCherry group (7.9 ± 2.072, *n* = 5) vs. the ChR2-mCherry group (62.86 ± 3.923, *n* = 5), showed the optogenetic stimulation significantly increase the cFos expression in orexin neurons. All data are expressed as mean ± SEM. Significance was analyzed using unpaired *t*-test between two groups. ****P < 0.0001.

After the successful viral expression, we validated the function of the optogenetic stimulation via the loose patch recording and cFos staining (Figure 5A). The orexin neurons expressing ChR2-mCherry in the acute brain slices showed reliable firing upon blue light (473 nm) photoactivation targeting the orexin cell bodies. We also confirmed that optogenetic stimulations excite orexin neurons reliably at different frequencies (1 Hz, 5 Hz, 10 Hz, and 20 Hz, 10 ms pulse width) (Figure 5B).

Compared with the mCherry group, optical stimulation remarkably elevated the percentage of double positive cell (cFos^+^+orexin^+^) among orexin neurons (orexin^+^) in the ChR2-mCherry group (mCherry group: 7.9 ± 2.072% vs. ChR2-mCherry group: 62.86 ± 3.923%; *n* = 5; *P* < 0.0001; Figures 5C,D). Our cFos staining confirmed that optogenetic stimulation can activate orexin neurons faithfully.

**FIGURE 6:**
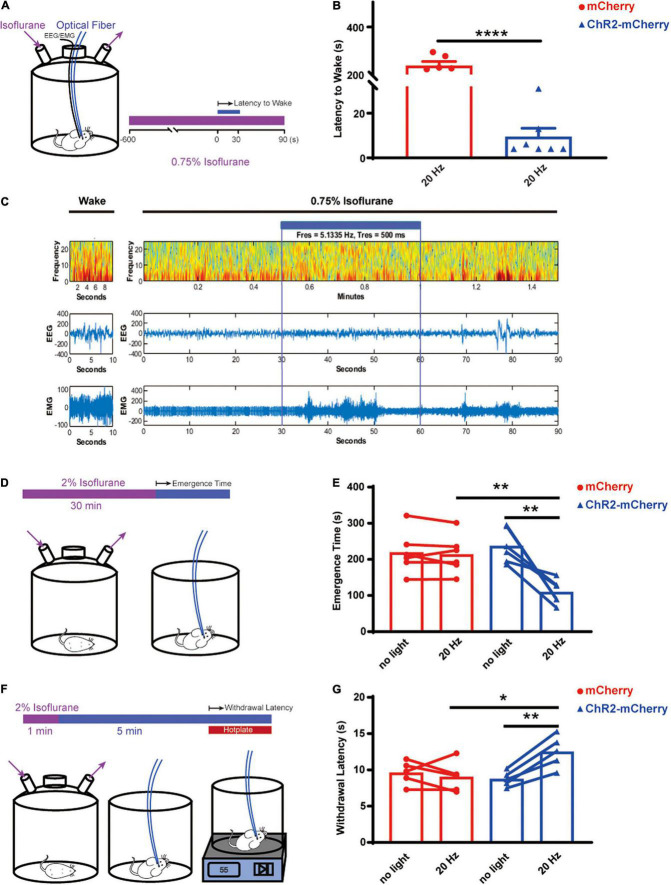
Optogenetic activation of orexin neurons facilitated arousal and pain tolerance. **(A)** Arousal test under 0.75% isoflurane. **(B)** Optogenetic stimulation elicited early arousal, while under 0.75% isoflurane, for the ChR2-mCherry group (9.429 ± 3.804, *n* = 7) but not the mCherry group (238.2 ± 17.42, *n* = 6). **(C)** EEG/EMG traces during wakefulness or under 0.75% isoflurane. Heat map showed the EEG spectra before, during and after optogenetic stimulation. **(D)** Emergence test after 2% isoflurane for 30 min. **(E)** Optogenetic stimulation significantly shortened the emergence time, for the ChR2-mCherry group (no light vs. 20 Hz, 237.2 ± 19.26 vs. 109.5 ± 13.59, *n* = 6) but not the mCherry group (no light vs. 20 Hz, 219.2 ± 24.15 vs. 213.8 ± 21.77, *n* = 6). Without stimulation, the ChR2-mCherry group has similar emergence time to the mCherry group. **(F)** Hotplate test. **(G)** Optogenetic stimulation increased the withdrawal latency to 55^°^C hotplate for the ChR2-mCherry group (no light vs. 20 Hz, 8.133 ± 0.5846 vs. 11.45 ± 1.185, *n* = 6) but not the mCherry group (no light vs. 20 Hz, 9.117 ± 0.663 vs. 8.767 ± 0.7775, *n* = 6). Without stimulation, the ChR2-mCherry group has similar withdrawal latency to the mCherry group. All data are expressed as mean ± S.E.M. Significance was analyzed using paired *t*-test within the same group of animals, and unpaired *t*-test between different groups. *P < 0.05, **P < 0.01, ****P < 0.0001.

To investigate the role of the orexin neurons in arousal and pain behavior in live animals, we first explored the arousal effect by optogenetically activating orexin neurons while the animals were under a light isoflurane anesthesia (Figure 6A). The results showed that optogenetic stimulation of orexin neurons can robustly induce arousal while the animals were under 0.75% isoflurane, whereas the control group completely stayed sedated (ChR2-mCherry group: 9.429 ± 3.804 s; *n* = 7 vs. mCherry group: 238.2 ± 17.42 s; *n* = 6; *P* < 0.0001; Figure 6B). The EEG/EMG recording showed the reversible changes of EEG spectrum upon the optogenetic stimulation (Figure 6C). When the mice were exposed to isoflurane at 1% or greater, neither group can be aroused upon stimulation.

We further asked whether the orexin activation can facilitate the emergence speed after 30 min of surgical level of isoflurane (2%) (Figure 6D). The paired *t*-test comparing the same animals showed the optogenetic activation significantly shortened the emergence time of the ChR2-mCherry group (no light: 237.2 ± 19.26 s vs. 20 Hz: 109.5 ± 13.59 s; *n* = 6; *P* = 0.005) but not the mCherry group (no light: 219.2 ± 24.15 s vs. 20 Hz: 213.8 ± 21.77 s; n = 6; P = 0.483; Figure 6E). Unpaired *t*-test showed the optogenetic activation significantly shortened the emergence time of the ChR2-mCherry group (mCherry group: 213.8 ± 21.77 s vs. ChR2-mCherry group: 109.5 ± 13.59 s; *n* = 6; *P* = 0.0023; Figure 6E).

Next, we tested the analgesic effect by activating orexin neurons through a 55^°^C hot plate test (Figure 6F). The paired *t*-test comparing the same animals showed that the optogenetic activation can lengthen the latency to withdraw significantly for the ChR2-mCherry group (no light: 8.133 ± 0.5846 s vs. 20 Hz: 11.45 ± 1.185 s; *n* = 6; *P* = 0.0159) but not the mCherry group (no light: 9.117 ± 0.663 s vs. 20 Hz: 8.767 ± 0.7775 s, *n* = 6; *P* = 0.5952; Figure 6G). Unpaired *t*-test displayed significantly longer latencies in the ChR2-mCherry group (mCherry group: 8.767 ± 0.7775 s vs. ChR2-mCherry group: 11.45 ± 1.185 s, *n* = 6; *P* = 0.0317; Figure 6G). It illustrated that optogenetic stimulation of the orexin neurons in the lateral hypothalamus increased the animal’s thermal pain tolerance.

## Discussion

Our results demonstrated not only novel orexin projection patterns but also robust behavioral response to optical manipulation of the orexin neuronal activities.

We found both anterograde AAV5 and retrograde AAVretro produced a robust, long-range expression of opsin in the orexin neuron terminals. The opsin staining showed high-resolution details of orexin fiber projections, due to a strong axonal membrane surface expression of the opsins. This is a fundamental step to establish an effective optogenetics technique to study neural circuits.

To confirm the projection pattern from the anterograde AAV5 expression, the retrograde AAVretro was injected into terminal areas. Strong signals were observed not only in the processes from the injection site to the orexin cell bodies, but also in the ones beyond the cell bodies to the other directions. It suggested, even though AAVretro is considered retrograde, the expression of opsin was not restricted in the processes from the injection sites to cell bodies (Tervo et al., 2016). Furthermore, we observed distinct patterns from different remote injections, suggesting that an individual orexin neuron selectively projects to multiple targets. It is a debatable topic regarding the heterogeneity of the orexin neuronal populations in the hypothalamus. While some studies supported that different local orexin populations from medial to lateral hypothalamus are involved in different functional roles (Harris and Aston-Jones, 2006), other studies suggested that the orexin neurons are more intermingled rather than discretely localized (González et al., 2012; Iyer et al., 2018).

The light sheet data from an intact brain allowed us to visualize 3D projections of orexin neurons. Compared to the conventional confocal microscope, the light sheet is faster to image a large sample such as a half mouse brain and imposes less phototoxicity (Tomer et al., 2014). However, it is more labor-intensive to clarify and stain the sample, and the resolution at a certain depth or angle may be affected by the lipid content (Gradinaru et al., 2018). As the confocal microscope produces 2D images with higher resolution, especially with the versatility to use different lenses, it is beneficial to combine both imaging technologies.

Our study revealed a number of novel orexin projection patterns which were not shown previously. Orexin projections into two neighboring nuclei, such as lateral habenula vs. medial habenula, substantia nigra pars compacta (SNc) vs. reticular part (SNr), are dramatically different. Lateral habenula is heavily innervated by orexin fibers in the thalamus, in sharp contrast to medial habenula with little orexin fibers. Although physically next to each other, lateral habenula and medial habenula seem to have distinct connections. Lateral habenula is known to be an essential player in regulating negatively motivated behavior through communicating with the rostromedial tegmental nucleus and all midbrain monoaminergic systems (Hu et al., 2020). Medial habenula, associated with addiction and mood disorders, releases acetylcholine, substance P, and glutamate, whereas lateral habenula mostly releases glutamate (Nakajo et al., 2020; Thomas et al., 2022). Our results suggested orexin might be involved in emotion and decision-making through the lateral habenula.

We found a dense projection to the SNc but not in the adjacent SNr in the midbrain. Substantia nigra is an important nucleus regulating motor and reward. It’s well known that Parkinson’s disease is mainly a motor disorder, which is linked to the loss of dopaminergic neurons in the SNc. A growing number of studies suggest that sleep dysfunction seems to precede the motor symptoms (Arnulf et al., 2000; Abbott et al., 2005). Comparing human brains from Parkinson’s disease patients with normal ones showed significant loss of orexin cells and the degree of loss is correlated with the severity of the disease. Our staining results reinforced that orexin circuits can be a potential therapeutic target for neurodegenerative diseases. Although both SNc and SNr have been shown to participate in sleep-wake control and motor behavior regulation, SNc and SNr are different histologically. SNc is involved in the modulation of sleep-wake through dopaminergic neurons (Yanagihara et al., 2020), while SNr is integrated with GABAergic neurons (Liu et al., 2020). More studies are needed to decipher the mechanisms underlying the selective distribution of orexin fibers.

Our results revealed that the programmable activation of the cell bodies of orexin neurons in lateral hypothalamus through optogenetic stimulation cannot only facilitate emergence after deep isoflurane anesthesia, but also directly reanimate the mice out of light isoflurane anesthesia. Due to the diurnal variation in the orexin neuronal activities on a 12-h light/dark cycle, we chose to conduct our experiments during the day when the orexin activity is low in mice in order to maximize the neuronal response to optogenetic stimulations (Estabrooke et al., 2001; Yoshida et al., 2001; McGregor et al., 2017). Compared to chemogenetics, optogenetics offers higher spatial and temporal resolution. Unlike the long-lasting effect of chemogenetic stimulation, the activation by optogenetics is short-lived and reversible (Vlasov et al., 2018). This technology is crucial for further study of the orexin-associated arousal circuits.

The exact mechanisms of entering and exiting from anesthesia are unclear. Previous studies have shown many reciprocal innervations between orexin neurons and other arousal nuclei in regulating not only sleep/wake cycle but also anesthesia emergence. Consistently, the mapping of orexin projections suggests that the arousal regulation during anesthesia by orexin is likely related to its extensive projections into the wake-promoting nuclei, including basal forebrain (Dong et al., 2009; Wang D. et al., 2021), VTA (Poller et al., 2013; Yanagihara et al., 2020), locus coeruleus (Horvath et al., 1999; Carter et al., 2013, 2012), tuberomammillary nucleus (Kiyashchenko et al., 2002; Torrealba et al., 2003; Saper et al., 2010), dorsal raphe (Saito et al., 2018; Yang et al., 2019), and sleep-promoting nucleus VLPO. The VLPO in the preoptic area of the anterior hypothalamus plays a major role in promoting sleep (Sherin et al., 1996; Liang et al., 2021). The majority of the VLPO neurons are GABAergic neurons that are active during sleep and innervate other arousal-related nuclei, including orexin (Vanini et al., 2020).

In addition to the arousal effect, we found that activating orexin neurons in the lateral hypothalamus can increase the animal’s tolerance to painful stimuli. Many of the orexin projected brain regions are also involved in the pain control, such as BST, central amygdala nucleus, dorsal nucleus raphe, laterodorsal tegmental area, lateral habenula, PAG, parabrachial nucleus, PVT, and VTA, which may underlie the analgesic effects by orexin neurons (Kooshki et al., 2020; Matini et al., 2020). Further work is needed to study the involvement of different nuclei in pain control.

## Data Availability Statement

The original contributions presented in the study are included in the article/Supplementary Material, further inquiries can be directed to the corresponding author/s.

## Ethics Statement

The animal study was reviewed and approved by the Institutional Animal Care Use Committee.

## Author Contributions

WZ, XX, K-XL, ZG, JF, and PB: conceptualization. XX, YC, ZG, and WZ: methodology and formal analysis. XX and WZ: writing—original draft preparation. WZ, ZG, JF, and PB: writing—review and editing. All authors have read and agreed to the published version of the manuscript.

## Conflict of Interest

The authors declare that the research was conducted in the absence of any commercial or financial relationships that could be construed as a potential conflict of interest.

## Publisher’s Note

All claims expressed in this article are solely those of the authors and do not necessarily represent those of their affiliated organizations, or those of the publisher, the editors and the reviewers. Any product that may be evaluated in this article, or claim that may be made by its manufacturer, is not guaranteed or endorsed by the publisher.
