# Supplementary material for: Neuroanatomical Basis for the Orexinergic Modulation of Anesthesia Arousal and Pain Control
Source: Front Cell Neurosci. 2022 Apr 26;16:891631. doi: 10.3389/fncel.2022.891631 (PMC9090436; doi:10.3389/fncel.2022.891631)
Supplement: Supplementary file 1 [file Data_Sheet_1.docx]

**Supplementary Materials:**

**Table S1**

| **Relative density of orexin fibers in mouse brain** | | | | |
| --- | --- | --- | --- | --- |
| Brain region | Fiber density* | |  |  |
| **Cortical plate** |  |  |  |  |
| [Isocortex](http://atlas.brain-map.org/atlas?atlas=1&plate=100960333) | + |  |  |  |
| [HPF, Hippocampal formation](http://atlas.brain-map.org/atlas?atlas=2&plate=100884129) | - |  |  |  |
| [OLF, Olfactory areas](http://atlas.brain-map.org/atlas?atlas=1&plate=100960333) | + |  |  |  |
| **Cortical subplate** |  |  |  |  |
| CLA, Claustrum | + |  |  |  |
| BLA, Basolateral amygdalar nucleus | + |  |  |  |
| BMA, Basomedial amygdalar nucleus | + |  |  |  |
| PA, Posterior amygdalar nucleus | + |  |  |  |
| **Striatum** |  |  |  |  |
| AAA, Anterior amygdalar area | ++ |  |  |  |
| ACB, Anterior amygdalar area | + |  |  |  |
| CEA, Central amygdalar nucleus | ++ |  |  |  |
| FS, Fundus of striatum | + |  |  |  |
| LS, Lateral septal nucleus | +++ |  |  |  |
| MEA, Medial amygdalar nucleus | +++ |  |  |  |
| OT, Olfactory tubercle | - |  |  |  |
| CP, Caudoputamen | - |  |  |  |
| [**Pallidum**](http://atlas.brain-map.org/atlas?atlas=2&plate=100884129) |  |  |  |  |
| BST, Bed nuclei of the stria terminalis | +++ |  |  |  |
| Gpe, Globus pallidus, external segment | + |  |  |  |
| Gpi, Globus pallidus, internal segment | ++ |  |  |  |
| MA, Magnocellular nucleus | ++ |  |  |  |
| MS, Medial septal nucleus | +++ |  |  |  |
| NDB, Diagonal band nucleus | +++ |  |  |  |
| SI, Substantia innominata | ++ |  |  |  |
| [**Thalamus**](http://atlas.brain-map.org/atlas?atlas=2&plate=100884129) |  |  |  |  |
| AD, Anterodorsal nucleus | + |  |  |  |
| CM, Central medial nucleus of the thalamus | +++ |  |  |  |
| IMD, Intermediodorsal nucleus of the thalamus | +++ |  |  |  |
| LH, Lateral habenula | +++ |  |  |  |
| PF, Parafascicular nucleus | + |  |  |  |
| PT, Parataenial nucleus | ++ |  |  |  |
| PVT, Paraventricular nucleus of the thalamus | +++ |  |  |  |
| RE, Nucleus of reuniens | +++ |  |  |  |
| RT, Reticular nucleus of the thalamus | +++ |  |  |  |
| SPFp, Subparafascicular nucleus, parvocellular part | + |  |  |  |
| **Hypothalamus** |  |  |  |  |
| AHN, Anterior hypothalamic nucleus | +++ |  |  |  |
| ARH, Arcuate hypothalamic nucleus | +++ |  |  |  |
| AVP, Anteroventral preoptic nucleus | +++ |  |  |  |
| AVPV, Anteroventral periventricular nucleus | +++ |  |  |  |
| DMH, Dorsomedial nucleus of the hypothalamus | +++ |  |  |  |
| LHA, Lateral hypothalamic area | +++ |  |  |  |
| LPO, Lateral preoptic area | +++ |  |  |  |
| ME, Median eminence | +++ |  |  |  |
| MEPO, Median preoptic nucleus | +++ |  |  |  |
| MPO, Medial preoptic area | +++ |  |  |  |
| MPN, Medial preoptic nucleus | +++ |  |  |  |
| MM, Medial mammillary nucleus | - |  |  |  |
| PH, Posterior hypothalamic nucleus | +++ |  |  |  |
| PMv, Ventral premammillary nucleus | +++ |  |  |  |
| PMd, Dorsal premammillary nucleus | +++ |  |  |  |
| PVH, Paraventricular hypothalamic nucleus | +++ |  |  |  |
| PVp, Periventricular hypothalamic nucleus, posterior part | +++ |  |  |  |
| RCH, Retrochiasmatic area | +++ |  |  |  |
| SCH, Suprachiasmatic nucleus | - |  |  |  |
| STN, Subthalamic nucleus | ++ |  |  |  |
| SUM, Supramammillary nucleus | +++ |  |  |  |
| TMd, Tuberomammillary nucleus, dorsal part | +++ |  |  |  |
| TMv, Tuberomammillary nucleus, ventral part | +++ |  |  |  |
| TU, Tuberal nucleus | +++ |  |  |  |
| VMH, Ventromedial hypothalamic nucleus | +++ |  |  |  |
| VLPO, Ventrolateral preoptic nucleus | +++ |  |  |  |
| ZI, Zona incerta | +++ |  |  |  |
| **Midbrain** |  |  |  |  |
| CLI, Central linear nucleus raphe | +++ |  |  |  |
| CS, Superior central nucleus raphe | ++ |  |  |  |
| CUN, Cuneiform nucleus | ++ |  |  |  |
| DR, Dorsal nucleus raphe | +++ |  |  |  |
| IC, Inferior colliculus | + |  |  |  |
| IF, Interfascicular nucleus raphe | ++ |  |  |  |
| IPN, Interpeduncular nucleus | - |  |  |  |
| MARN, Magnocellular reticular nucleus | ++ |  |  |  |
| MPT, Medial pretectal area | ++ |  |  |  |
| MRN, Midbrain reticular nucleus | +++ |  |  |  |
| NPC, Nucleus of the posterior commissure | ++ |  |  |  |
| PAG, Periaqueductal gray | +++ |  |  |  |
| PPT, Posterior pretectal nucleus | + |  |  |  |
| PRC, Precommissural nucleus | ++ |  |  |  |
| RL, Rostral linear nucleus raphe | +++ |  |  |  |
| RR, Midbrain reticular nucleus, retrorubral area | + |  |  |  |
| SCm, Superior colliculus, motor related | + |  |  |  |
| SCs, Superior colliculus, sensory related | - |  |  |  |
| SNc, Substantia nigra, compact part | +++ |  |  |  |
| SNr, Substantia nigra, reticular part | + |  |  |  |
| VTA, Ventral tegmental area | +++ |  |  |  |
| **Pons** |  |  |  |  |
| DTN, Dorsal tegmental nucleus | ++ |  |  |  |
| IRN, Intermediate reticular nucleus | + |  |  |  |
| LC, Locus coeruleus | +++ |  |  |  |
| LDT, Laterodorsal tegmental nucleus | +++ |  |  |  |
| NI, Nucleus incertus | ++ |  |  |  |
| PB, Parabrachial nucleus | +++ |  |  |  |
| PG, Pontine gray | - |  |  |  |
| PRNc, Pontine reticular nucleus, caudal part | + |  |  |  |
| PRNr, Pontine reticular nucleus | + |  |  |  |
| SOC, Superior olivary complex | + |  |  |  |
| TRN, Tegmental reticular nucleus | + |  |  |  |
| **Medulla** |  |  |  |  |
| MY, Medulla | + |  |  |  |

*Rating scale: +++, high fiber density; ++, medium fiber density; +, low fiber density; and -, insignificant number of fibers.


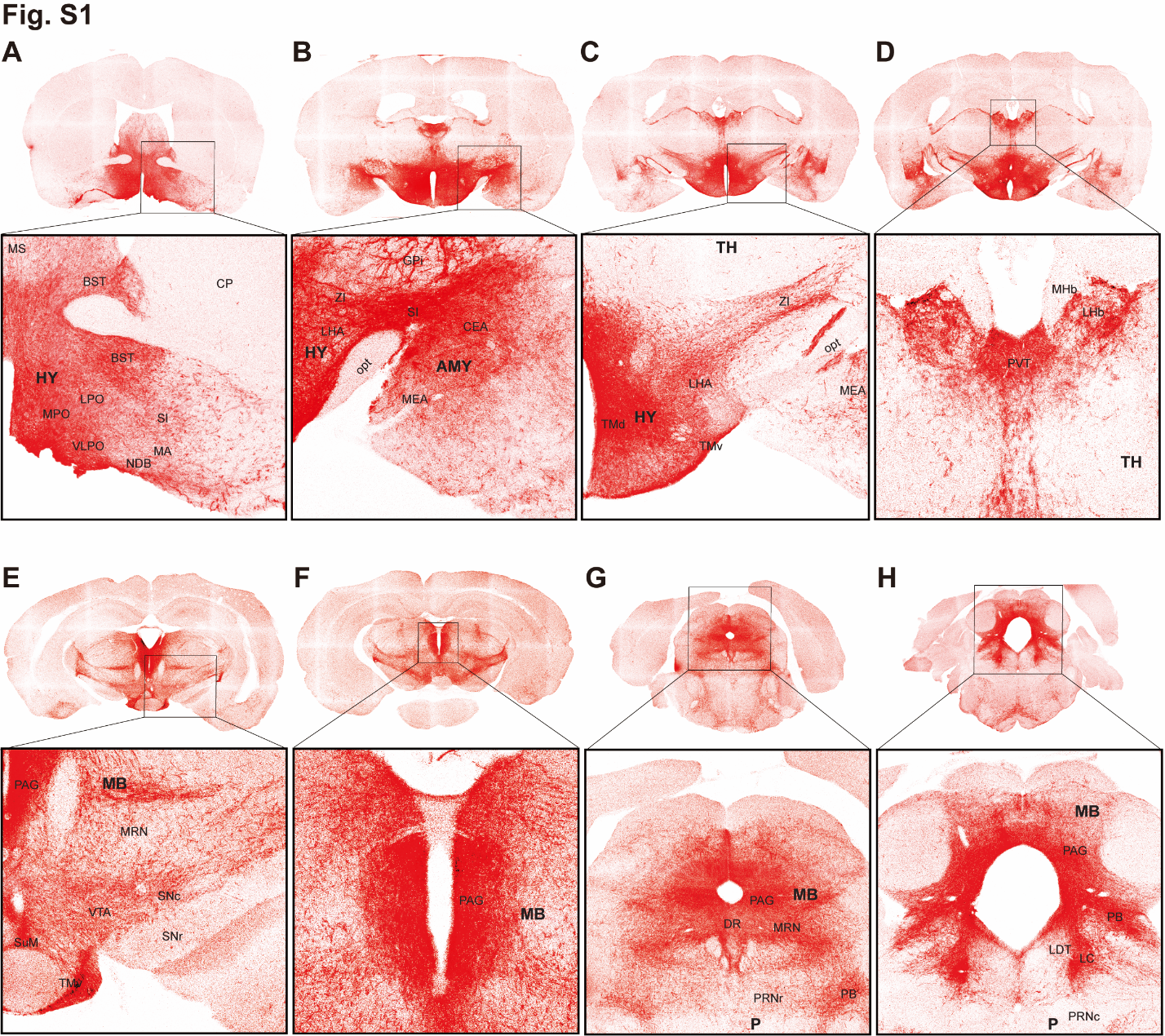


**Fig. S1. Orexin projections to the nuclei related to sleep-wake and pain control.** Orexin-Cre mice were injected with AAV_retro_-DIO-ChR2-mCherry into LHA. The immunostaining of mCherry highlighted the projections of orexin fibers to the nuclei related to sleep-wake and pain control. AMY, amygdala; BST, bed nuclei of the stria terminalis; CEA, central amygdalar nucleus; CP, caudoputamen; DR, dorsal nucleus raphe; GPi, globus pallidus, internal segment; HY, hypothalamus; LC, locus coeruleus; LDT, laterodorsal tegmental nucleus; LHA, lateral hypothalamic area; LHb, lateral habenula; LPO, lateral preoptic area; MA, magnocellular nucleus; MB, midbrain; MEA, medial amygdalar nucleus; MHb, medial habenula; MPO, medial preoptic area; MS, medial septal nucleus; MRN, midbrain reticular nucleus; NDB, diagonal band nucleus; opt, optic tract; P, pons; PAG, periaqueductal gray; PB, parabrachial nucleus; PVT, paraventricular nucleus of the thalamus; SI, substantia innominata; SNc, substantia nigra, compact part; SNr, substantia nigra, reticular part; SUM, supramammillary nucleus; TH, thalamus; TMd, tuberomammillary nucleus, dorsal part; TMv, tuberomammillary nucleus, ventral part; VLPO, ventrolateral preoptic nucleus; VTA, ventral tegmental area; ZI, zona incerta.

**
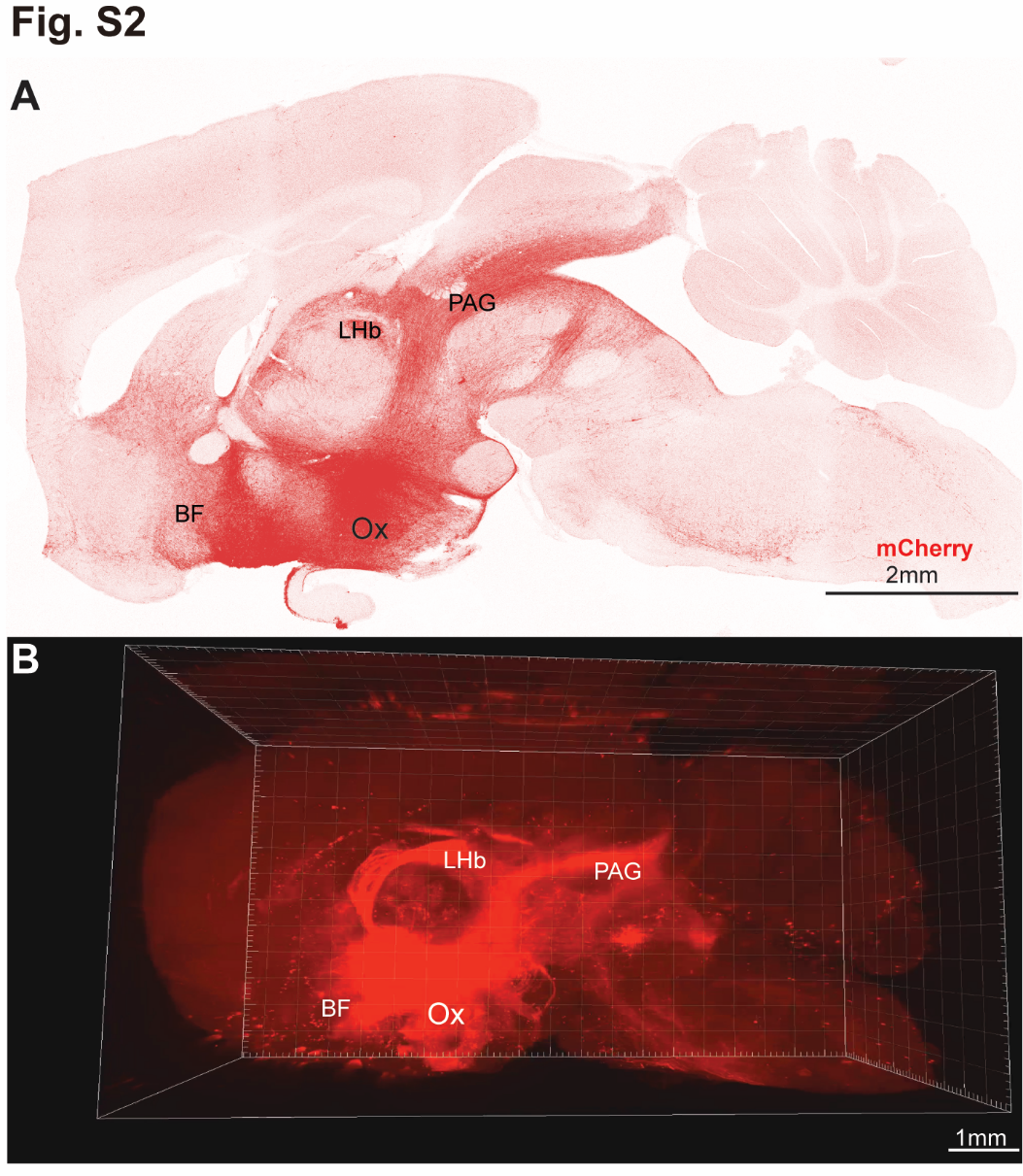
**

**Fig. S2. Broad projections of orexin circuit with mCherry staining shown by confocal image (A) and light sheet image (B).** Orexin-Cre mice were injected with AAV_retro_-DIO-ChR2-mCherry into LHA. 3D lightsheet rendering is visualized with Imaris program. BF, basal forebrain; LHb, lateral habenula; PAG, periaqueductal gray.

**Supplementary Video**

**Video S1.** **Light sheet video of orexin projections in the mouse brain.** Orexin-Cre mice were injected with AAV_retro_-DIO-ChR2-mCherry into the lateral hypothalamus. A brain hemisphere was used for light sheet imaging after clarification and staining with anti-mCherry. Light sheet images were taken with ZEISS Lightsheet Z.1. Tile scans are assisted with the setup of a tiling experiment with Zen (ZEISS) for light-sheet Z.1 software. Tiles were stitched and 3D video was generated by Imaris software. The video showed the broad 3D projections of orexin neurons in the mouse brain.
